# Effect of virtual reality training to enhance laparoscopic assistance skills

**DOI:** 10.1186/s12909-023-05014-5

**Published:** 2024-01-04

**Authors:** Xiuwen Chen, Peng Liao, Shiqing Liu, Jianxi Zhu, Abdullah Sultan Abdullah, Yao Xiao

**Affiliations:** 1 Teaching and Research Section of Clinical Nursing, Xiangya Hospital, Central South University, Changsha, China; 2 Xiangya School of Nursing, Xiangya Hospital, Central South University, Changsha, China; 3 Department of Respiratory Medicine, Xiangya Hospital, Central South University, Changsha, China; 4 Hunan Key Laboratary of Aging Biology, Xiangya Hospital, Central South University, Changsha, 410008 China; 5 Department of General Surgery, Xiangya Hospital, Central South University, Changsha, China; 6 International Joint Research Center of Minimally Invasive Endoscopic Technology Equipment & Standards, Xiangya Hospital, Central South University, Changsha, China; 7 National Clinical Research Center for Geriatric Disorders, Xiangya Hospital, Central South University, Changsha, China

**Keywords:** Virtual reality, Laparoscopic operation, Operating room nurses, Simulation training

## Abstract

**Background:**

While laparoscopic assistance is often entrusted to less experienced individuals, such as residents, medical students, and operating room nurses, it is important to note that they typically receive little to no formal laparoscopic training. This deficiency can lead to poor visibility during minimally invasive surgery, thus increasing the risk of errors. Moreover, operating room nurses and medical students are currently not included as key users in structured laparoscopic training programs.

**Objectives:**

The aim of this study is to evaluate the laparoscopic skills of OR nurses, clinical medical postgraduate students, and residents before and after undergoing virtual reality training. Additionally, it aimed to compare the differences in the laparoscopic skills among different groups (OR nurses/Students/Residents) both before and after virtual reality training.

**Methods:**

Operating room nurses, clinical medical postgraduate students and residents from a tertiary Grade A hospital in China in March 2022 were selected as participants. All participants were required to complete a laparoscopic simulation training course in 6 consecutive weeks. One task from each of the four training modules was selected as an evaluation indicator. A before-and-after self-control study was used to compare the basic laparoscopic skills of participants, and laparoscopic skill competency was compared between the groups of operating room nurses, clinical medical postgraduate students, and residents.

**Results:**

Twenty-seven operating room nurses, 31 clinical medical postgraduate students, and 16 residents were included. The training course scores for the navigation training module, task training module, coordination training module, and surgical skills training module between different groups (operating room nurses/clinical medical postgraduate/residents) before laparoscopic simulation training was statistically significant (*p* < 0.05). After laparoscopic simulation training, there was no statistically significant difference in the training course scores between the different groups. The surgical level scores before and after the training course were compared between the operating room nurses, clinical medical postgraduate students, and residents and showed significant increases (*p* < 0.05).

**Conclusion:**

Our findings show a significant improvement in laparoscopic skills following virtual surgery simulation training across all participant groups. The integration of virtual surgery simulation technology in surgical training holds promise for bridging the gap in laparoscopic skill development among health care professionals.

**Supplementary Information:**

The online version contains supplementary material available at 10.1186/s12909-023-05014-5.

## Introduction

Compared to traditional open surgery, laparoscopic techniques have been developed to accelerate postoperative recovery, reduce surgical trauma, relieve pain, shorten the hospital stay, and improve cosmetic outcomes [1]. Fundamentally, laparoscopy involves the use of lenses, optical fibres, and related devices to display an image of the abdominal cavity onto a display screen. Surgeons then perform operations within this three-dimensional space, guided by the image displayed on the screen. The distinctive techniques needed for laparoscopic surgery clearly differ from those needed for traditional open surgery, leading to a steep learning curve [2]. This challenge is particularly pronounced for operating room nurses. Despite the transformative impact of laparoscopic techniques on surgical procedures, these nurses did not receive any education on laparoscopic surgeries during their academic training. Consequently, upon entering the field, they encounter entirely novel laparoscopic procedures, which pose considerable challenges to their learning process.

In laparoscopic surgery, tasks such as camera navigation and basic laparoscopic procedures are frequently delegated to residents or medical students with limited experience [3]. In some cases, operating room (OR) nurses also shoulder these responsibilities, especially in relatively straightforward procedures such as laparoscopic cholecystectomy or during periods of understaffing, such as during the COVID-19 outbreak [4]. However, regardless of whether they are residents, medical students, or OR nurses, they often receive limited to no formal laparoscopic training as part of their undergraduate medical education [5]. This lack of formal training is rare in current surgical practice, in which minimally invasive techniques are being increasingly used. Consistent evidence indicates that lack of experience is a significant contributor to the incidences of errors and complications in surgery [6, 7]. Consequently, surgery trainees are being progressively advised to undergo virtual reality simulation training before engaging in minimally invasive surgery procedures.

Recently, laparoscopic surgery simulators have been increasingly used across various domains of medical training [8]. These simulators utilize 3D graphics modelling and dynamic feedback technology to recreate the hardware components and microanatomy for laparoscopic surgery, mirroring the procedures encountered in clinical practice [9]. Students can engage in instrument manipulation and conduct relevant surgical drills within this virtual environment. Repeated surgical simulations enhance efficiency and accuracy and reduce clinical surgery risks. There is a growing body of evidence supporting the notion that simulation training can enhance surgical performance among residents [10, 11]. Presently, structured training curricula for laparoscopic surgery, both within and outside the operating room, exist, and studies have highlighted the significant benefits of virtual simulation training for facilitating the mastery of the learning curve and enhancing surgeons’ competency [12]. However, research analysing the impact of virtual reality simulation is predominantly limited to residents and medical students, with scarce data available on laparoscopic training for OR nurses.

In theory, OR nurses may possess a practical advantage over medical students, as they often observe and collaborate in laparoscopic surgeries, thereby gaining familiarity with surgical instruments [4]. Therefore, this study is designed with two primary objectives: first, to assess the laparoscopic skills of OR nurses, clinical medical postgraduate students, and residents before and after undergoing virtual reality training; second, to compare the differences in laparoscopic skills among various groups (OR nurses/Students/Residents) both before and after virtual reality training.

## Materials and methods

### Study population

OR nurses, clinical medical postgraduate students and residents of a tertiary Grade A hospital in China in March 2022 were enrolled as the study population. Informed consent was obtained from all individual participants included in the article. The inclusion criteria for OR nurses were as follows: (1) have obtained a nurse qualification certificate; (2) have worked in the OR within 3 years; (3) have engaged in clinical nursing work; and (4) have an understanding or familiarity with laparoscopic procedures, techniques, or principles. The postgraduate students in clinical medicine were third-year postgraduate medical students who were in the clinical rotation during their final year. The residents were surgical residents who had worked for less than two years. None of the participants had previously participated in laparoscopic simulation training. This study was approved by the Ethics Committee of Xiangya Hospital, Central South University (registry number 2022100820).

### Study design

We conducted a before-and-after self-control study to assess and compare the baseline laparoscopic surgical skills of three groups: OR nurses, clinical medical postgraduate students, and residents. In this study, each participant served as their own control, allowing us to meticulously assess and measure their individual skill levels both before and after they underwent training with the surgery simulator. By comparing the posttraining results with their initial baseline performance, we could quantify the specific skill enhancements gained through simulator-based training. Additionally, we performed between-group comparisons to analyse the laparoscopic adjunct competencies among these three groups of participants. This comparative analysis provided valuable insights into how each group responded to the training and whether any significant differences or trends emerged in skill development among these distinct categories of participants.

### Sample size

We conducted a before-and-after self-control study with a test level of ɑ = 0.05 and two-sided probability values of *P* values. The sample size is calculated as follows:

1 $$\begin{array}{l}N=\frac{{2SD}^{2}{\left({Z}_{\alpha /2}+{Z}_{\beta }\right)}^{2}}{{d}^{2}}\\ N=\frac{{2\times 8.402}^{2}\times {\left(1.960\times 0.842\right)}^{2}}{{11.600}^{2}}\approx 9\end{array}$$

*N* = Sample size

*SD* – Standard deviation of difference between experimental group and control group = 8.402 (From previous studies [13])

*Z*_*α*/2_ = *Z*_0.05/2_ = *Z*_0.025_ = 1.960 (From Z-value table) at type 1 error of 5%

Z_β_ = Z_0.20_ = 0.842 (From Z-value table) at 80% power

*d* = effect size = difference between mean values = 11.600 (from previous studies [13])

According to the sample size, it is estimated that at least 9 participants should be included in each group in this study. To prevent the withdrawal of participants during the study, the sample size was kept small, and the withdrawal rate was expected to be no more than 20%. This study will include more than 12 participants in each group.

### Simulator model

**Fig. 1:**
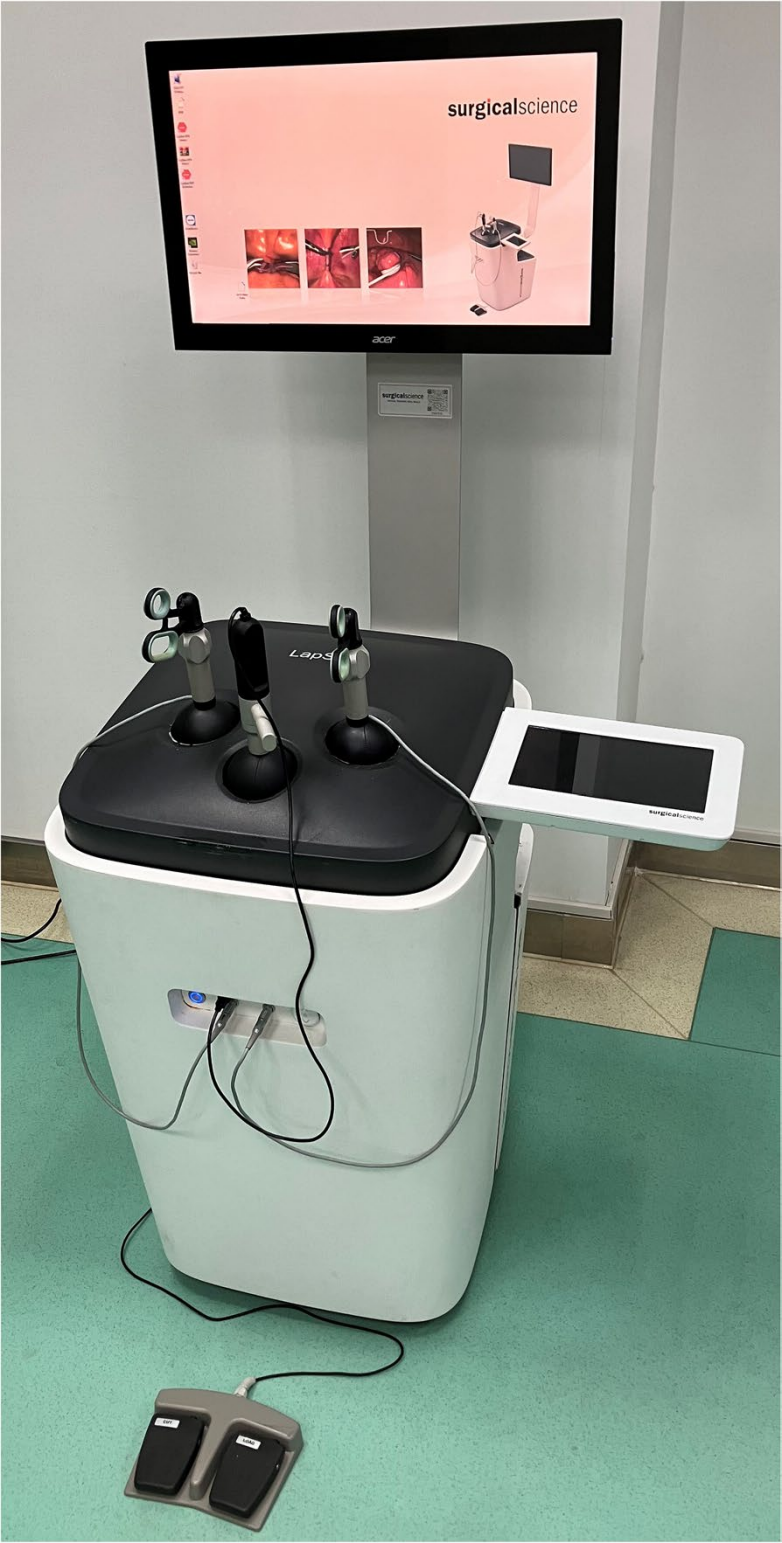
The laparoscopic simulation training system

The laparoscopic simulation training system is shown in Fig. 1. The system hardware includes a dual touch screen with a 27-inch main monitor and a 10-inch touch screen control monitor equipped with an integrated wireless mouse keyboard and a foot pedal. Such hardware can be used to facilitate electrocutting and electrocoagulation during surgery. The magnetic induction force feedback can automatically adjust the feedback strength according to the virtual surgical instruments touching virtual objects or human tissues; the left- and right-hand operated instruments can simulate various real instruments needed in the surgical process: grasping forceps, electrodes, stapler, ultrasonic knife, etc. They can be replaced by rotating the handle. The virtual camera system can realize 360-degree rotation, focal length adjustment, light intensity adjustment, angle adjustment and anatomical navigation. The software configuration mainly includes Camera navigation, Instrument navigation, Coordination, Grasping, Cutting, Suturing, Catheter Insertion, Clip Applying, Lifting & Grasping, Handling Intestines, Fine Dissection, Seal & Cut, Precision & Speed, Peg Transfer, Pattern Cutting, Ligating Loop, Wireloop, etc.

### Intervention procedure

Participants were enrolled in a laparoscopic simulation training course for 6 weeks, with a structured session held weekly for a total of 12 h of instruction. The training approach is the integration of PowerPoint presentations with simulated hands-on exercises. Sessions typically commenced with a 30-min theoretical lecture via PowerPoint, followed by a 90-min practical hands-on training. Assessments were conducted after each teaching phase; participants who did not meet the proficiency level were taken back to the previous step to reinforce understanding of the preceding content until they achieved the needed proficiency level to progress to the next stage. The initial performance of the participants was assessed before the intervention was implemented, and the participants’ operative performance was assessed posttraining.

The training course included preoperative preparation for laparoscopic surgery, pegboard movement training, intraoperative precautions for laparoscopic surgery, shape-specific tailoring training, management of postoperative complications of laparoscopic surgery, endoscopic loop coil training, basic laparoscopic operations, laparoscopic suture knotting training, simulation training of cholecystectomy, skills operation instruction, and video explanation of laparoscopic surgery (Table 1).

**Table 1:** Laparoscopic simulation training course setting

Time	Content	Form	Period
Week1	Preoperative preparation for laparoscopic surgery Pegboard movement training	Powerpoint lecture Operation training	2 h
Week2	Intraoperative precautions for laparoscopic surgery Shape-specific tailoring training	Powerpoint lecture Operation training	2 h
Week3	Management of postoperative complications of laparoscopic surgery Endoscopic loop coils training	Powerpoint lecture Operation training	2 h
Week4	Basic laparoscopic operations Laparoscopic suture knotting training	Powerpoint lecture Operation training	2 h
Week5	Simulation training of cholecystectomy Skills operation instruction	Surgery video display Simulated Surgery	2 h
Week6	Video explanation of laparoscopic surgery Skills operation instruction	Powerpoint lecture Operation training	2 h

### Assessment of outcomes

**Fig. 2:**
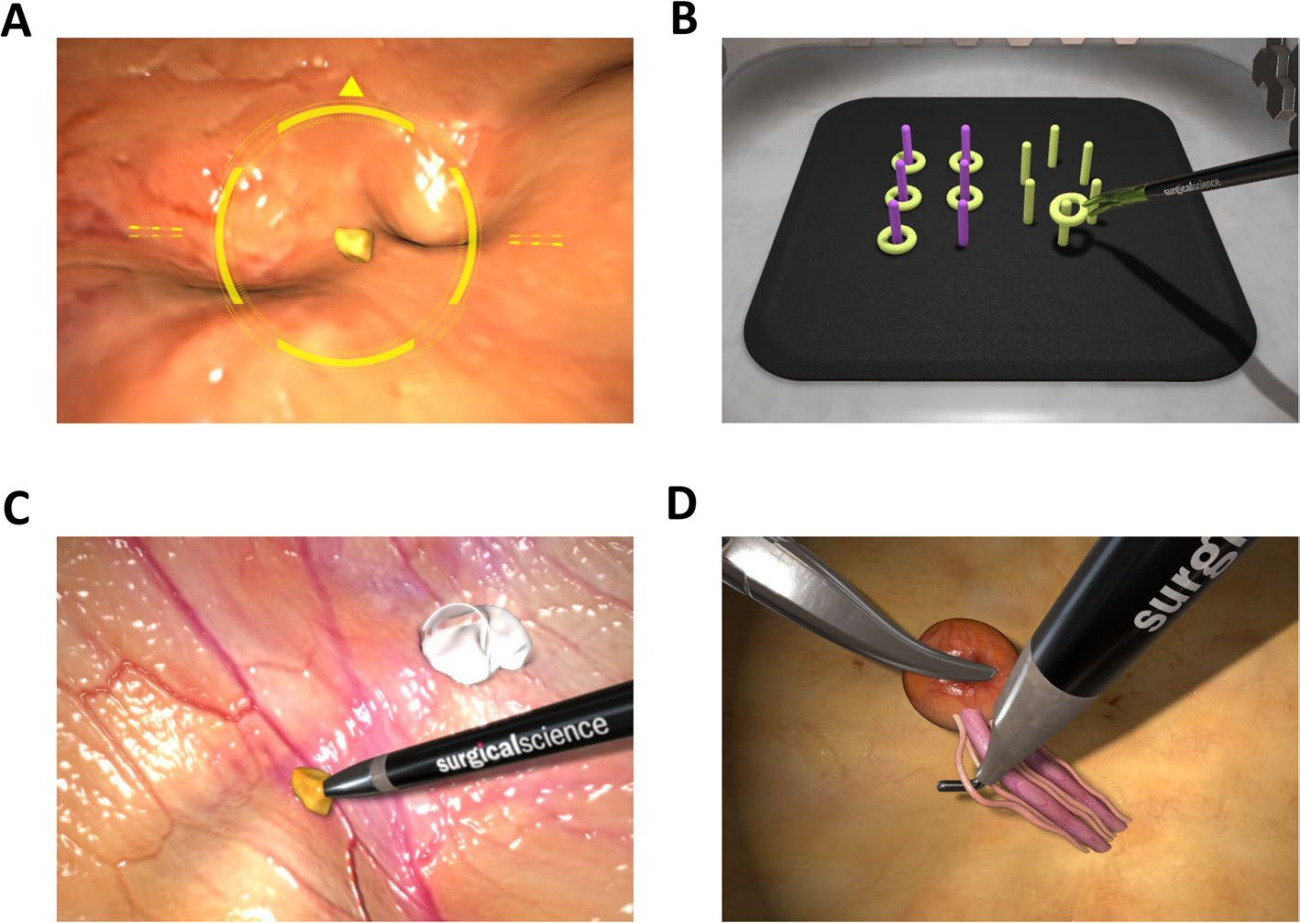
**A** Camera navigation; **B** Task training module: Peg Transfer; **C** Coordination training module: Coordination; **D** Surgical skills training module: Fine dissection

One task from each of the four training modules of the simulator was selected as evaluation indicators as follows: Navigation training module: Camera navigation (Fig. 2A); Task training module: Peg Transfer (Fig. 2B); Coordination training module: Coordination (Fig. 2C); Surgical skills training module: Fine dissection (Fig. 2D). Participants were evaluated by the simulator for the above indicators, and then a score was calculated on a scale of 0–100 (Table S1-4). Higher scores indicate a higher skill level.

### Quality control

Considering that there may be bias in the following five aspects in randomized controlled trials, namely, bias in the process of randomization, bias in deviation from the established intervention measures, bias in missing outcome data, bias in outcome measurement and bias in selective reporting of results, the aim of this study was to reduce the bias or the influence of subjective factors through several quality control methods in the following four aspects. The process of randomization was excluded due to the before-and-after self-control study design:

1. Intervention measures:
  First, ensure that the experimental data and the control data were from the corresponding treatment, respectively. Before measuring the data before intervention, ensure that the participants are not exposed to the relevant information of the training course and the laparoscopic simulation training system, and all enrolled participants were required to complete the entire training to ensure that the experimental data and the control data were from the corresponding treatment.
  Second, the use of quantitative, objective measures to assess outcomes, rather than nonquantitative, subjectively reported measures, ensured that the effect of the intervention was not affected by the subjective factors of the participants.
  In addition, ensure consistency in the intervention process and course instructors, and for who the identity of the participants was concealed. The same intervention process was used for all participants. The same course was taught by the same instructors, and the identity of the participants was concealed to ensure that the subjective factors of the course instructors did not affect the implementation of the intervention among the nurse group, the student group and the resident group.
2. Missing outcome data: The outcome data of almost all participants were collected. Both the outcome data and the control data of the participants who did not finish the entire training were excluded from the study to balance the missing data between the control data and the intervention data.
3. Outcome measurement: The same criteria and procedures were employed for collecting scores from participants before and after the intervention via computer.
4. Selective reporting of results: The selection of outcome evaluation indicators, statistical analysis methods and test levels are determined before the implementation of the study to ensure that the implementer will not choose evaluation indicators and analysis methods according to the merits of the results during data analysis to avoid the influence of the implementer’s subjective factors on the study.

Additionally, our study underwent peer review and team discussions, incorporating feedback and suggestions from multiple professionals to refine and enhance our course design and the implementation evaluation process.

### Statistical analysis

SPSS 22.0 software was used for data entry and statistical analysis. The measurement data were described by mean ± standard deviation (SD) and statistically analysed by t test or ANOVA; the count data were described by frequency and percentage and statistically analysed by χ^2^ test, Fisher’s exact probability or rank sum test. Statistical inference was performed at a test level of ɑ = 0.05, and *P* values were taken as two-sided probability values, with *P* < 0.05 being a statistically significant difference in comparison.

## Results

### Demographic characteristics

A total of 27 OR nurses, 31 clinical medical postgraduate students, and 16 residents were included. One operating room nurse was excluded because of studying abroad without completing the laparoscopic simulation training course. There were 21 female and 6 male OR nurses, aged 20 to 26 (22.44 ± 1.502) years, who had worked in the OR for 9 to 32 (20.33 ± 8.138) months, and all had a bachelor’s degree or higher (Table 2).

**Table 2:** General information of the study subjects

Characteristics	OR nurses	Students	Residents
Group size	27	31	16
Age, years (mean ± SD)	22.4 ± 1.5	24.8 ± 1.1	29.9 ± 1.7
Working in OR, months (mean ± SD)	20.3 ± 8.1	13.7 ± 4.1	16.3 ± 5.4
Gender (n, %)			
Male	6(22.22)	18(58.06)	12(75.00)
Female	21(77.78)	13(41.94)	4(25.00)
Hand dominance (n, %)			
Right	27(100.00)	29(93.55)	15(93.75)
Left	0(0.00)	2(6.45)	1(6.25)
Laparoscopic experience (1st assistance) (n, %)			
Yes	25(92.59)	27(87.10)	16(100.00)
No	2(7.41)	4(12.90)	0(0.00)
Number of assisted laparoscopic procedures (mean ± SD)	101.2 ± 60.9	77.2 ± 32.6	173.2 ± 101.7

### Initial performance level

The comparison of training course scores for the navigation training module, task training module, coordination training module, and surgical skills training module between different groups (OR nurses/Students/Residents) before laparoscopic simulation training was statistically significant (*p* < 0.05), and residents had the highest scores (Table 3). In addition, there was no significant difference in the training task scores between the nurses and medical students (*p* > 0.05).

**Table 3:** Comparison of initial training course scores between different groups (OR nurses/Students/Residents)

		OR nurses	Students	Residents	*F*	*P*
Navigation training module	Camera navigation	65.07 ± 10.118	68.97 ± 9.676	86.94 ± 4.553	31.476	< 0.001
Task training module	Peg transfer	67.30 ± 7.482	68.65 ± 6.264	79.13 ± 7.813	15.742	< 0.001
Coordination training module	Coordination	69.04 ± 6.897	68.68 ± 7.782	75.88 ± 5.644	6.249	0.003
Surgical skills training module	Fine dissection	67.93 ± 5.327	72.58 ± 6.054	81.06 ± 7.113	23.719	< 0.001

### Operative performance after training course

#### Before and after self-control

The scores of operating room nurses, medical postgraduates and residents after completing the training courses were significantly higher than the initial performance, and the difference was statistically significant (*p* < 0.05) (Table 4).

**Table 4:** Comparison of surgical level scores before and after the training course among OR nurses, clinical medical postgraduate students, and residents

			Initial Performance	Performance After Training Course	t	*P*
OR nurses	Navigation training module	Camera navigation	65.07 ± 10.118	90.26 ± 5.223	-14.854	< 0.001
	Task training module	Peg transfer	67.30 ± 7.482	86.96 ± 6.358	-14.099	< 0.001
	Coordination training module	Coordination	69.04 ± 6.897	89.37 ± 4.289	-15.226	< 0.001
	Surgical skills training module	Fine dissection	67.93 ± 5.327	88.26 ± 4.579	-17.590	< 0.001
Students	Navigation training module	Camera navigation	68.97 ± 9.676	91.77 ± 5.959	-14.765	< 0.001
	Task training module	Peg transfer	68.65 ± 6.264	88.81 ± 4.324	-15.707	< 0.001
	Coordination training module	Coordination	68.68 ± 7.782	88.71 ± 4.043	-14.300	< 0.001
	Surgical skills training module	Fine dissection	72.58 ± 6.054	90.87 ± 4.113	-14.333	< 0.001
Residents	Navigation training module	Camera navigation	86.94 ± 4.553	93.50 ± 4.487	-4.565	< 0.001
	Task training module	Peg transfer	79.13 ± 7.813	90.50 ± 3.967	-6.989	< 0.001
	Coordination training module	Coordination	75.88 ± 5.644	92.00 ± 5.342	-8.441	< 0.001
	Surgical skills training module	Fine dissection	81.06 ± 7.113	90.56 ± 4.816	-6.219	< 0.001

#### Control among groups

The comparison of the training course scores in the navigation training module, task training module, coordination training module, and surgical skills training module between different groups (OR nurses/Students/Residents) after laparoscopic simulation training was not statistically significant (*p* > 0.05) (Table 5).

**Table 5:** Comparison of training course scores between different groups (OR nurses / clinical medical postgraduate / Residents) after training

		OR nurses	Students	Residents	*F*	*P*
Navigation training module	Camera navigation	90.26 ± 5.223	91.77 ± 5.959	93.50 ± 4.487	1.833	0.167
Task training module	Peg transfer	86.96 ± 6.358	88.81 ± 4.324	90.50 ± 3.967	2.511	0.088
Coordination training module	Coordination	89.37 ± 4.289	88.71 ± 4.043	92.00 ± 5.342	2.996	0.056
Surgical skills training module	Fine dissection	88.26 ± 4.579	90.87 ± 4.113	90.56 ± 4.816	2.757	0.070

## Discussion

Laparoscopic training based on a virtual surgical simulation system improves the laparoscopic skills of residents, clinical medical postgraduate students, and OR nurses.

In this study, there was a significant improvement in the laparoscopic surgical skills of residents, postgraduate students, and OR nurses after undergoing laparoscopic training using a virtual surgical simulation system. This finding aligns with the research by Kojima et al. [8]. Several studies have consistently shown that the integration of laparoscopic virtual simulation systems into surgical training has numerous benefits, including shortening the learning curve associated with laparoscopic surgery, enhancing surgeons’ suturing and tying skills, shortening surgical procedure durations, and decreasing the incidence of perioperative complications [14–16]. It is important to note that residents, postgraduate students, and OR nurses often receive limited or no formal laparoscopic training as part of their medical education [5]. This gap in laparoscopic skill development poses a well-recognized challenge in medical training programs. However, the introduction of virtual laparoscopic training technology has effectively addressed this issue by providing these health care professionals with a more accessible, intuitive, and cost-effective means of refining their skills. This innovative approach allows users to interact with virtual instruments, perform surgical procedures, and receive immediate feedback on their performance. The implications are significant, particularly for less-experienced individuals such as residents, postgraduate students, and nurses. Practising in a simulated, risk-free environment enables trainees to explore various scenarios without apprehension, ultimately reducing the time needed to master complex surgical procedures. Moreover, simulation-based training empowers trainees to adapt to the growing complexity and diversity of surgical procedures.

### Laparoscopic training based on a virtual surgical simulation system equips OR nurses with basic laparoscopic skills

Camera navigation and basic laparoscopic operation are key responsibilities of laparoscopic surgical assistants. The performance of the assistant may affect the surgical procedure [17, 18]. Previous studies on laparoscopic training via virtual simulation systems have largely focused on residents, and few studies have evaluated the competency of clinical medical postgraduate students and OR nurses, who perform most of these basic laparoscopic operations such as camera navigation in practice, especially when there is a lack of human resources due to public health emergencies such as the coronavirus pneumonia outbreak. New operating room nursing graduates are rarely exposed to laparoscopic surgery because the curriculum at school is mainly based on theoretical knowledge. Therefore, it is necessary to conduct laparoscopic training based on a virtual surgical simulation system. The results of this study showed that after laparoscopic training based on a virtual surgical simulation system, there was no statistically significant difference between the scores of basic laparoscopic operation skills of OR nurses and those of clinical medical postgraduate students and residents, while before the training, their scores were much lower than those of residents. Therefore, this study suggests that laparoscopic training based on a virtual surgical simulation system can be incorporated into the induction program for OR nurses to improve their job competency. Additionally, to our knowledge, there are limited studies investigating the efficacy of laparoscopic virtual training courses for OR nurses. Although the European Institute of Telesurgery offers laparoscopic training courses for OR nurses, these programs primarily concentrate on imparting relevant knowledge about laparoscopic surgery and equipment, offering only a brief introduction to practical handling on a box trainer. Our research can provide greater insights because virtual reality training enhanced the laparoscopic skills of OR nurses.

With continuous improvements in health care, the demand for well-educated nurses has greatly increased, and the numbers of operating room nurses and other nurses are also increasing [19, 20]. Therefore, the key to effective nursing education is the optimization of the teaching process and the cultivation of nurses that demonstrate professional quality or professional skills in the operating room [21]. Operating room emergencies, strict aseptic requirements, and strong specialization also put forward high requirements for the workload of operating room nurses [22]. In addition, with the continuous increase in endoscopic surgery and robotic surgery, new requirements and challenges have been put forward for the professional collaboration among operating room nurses [23, 24]. However, at present, the aim of training methods for operating room nurses is the improvement of traditional teaching models, such as hierarchical teaching models and situational simulation training models, and virtual reality simulation training models for surgery training are limited in terms of availability [13, 25]. Under the traditional teaching mode, operating room nurses are not encouraged to actively think and solve problems and acquire knowledge but rather to generally implement medical orders mechanically [13]. They usually cannot adapt to their internship quickly, often feel afraid, nervous or confused, and poorly understand the working conditions in the operating room [26]. An increasing number of studies are reporting the integration of precision medicine, artificial intelligence and virtual reality, and new technologies are needed to improve the quality and level of medical teaching [27, 28]. The application of virtual simulation technology, artificial intelligence and automated learning in various fields has promoted the calculation and comprehensive evaluation of multivariate datasets and improved the efficiency and quantification of medical practice evaluation and findings [29, 30]. In this study, a laparoscopy virtual simulation system was used to train operating room nurses, and the research results were consistent with the above studies [27, 28]. Therefore, the development of laparoscopic virtual simulation system training not only provides a new perspective for the training of operating room nurses but also enables the automation and informatization of traditional forms of teaching, which is also worthy of clinical promotion.

## Brief summary

In this study, the application of laparoscopic training via a virtual surgical simulation system notably enhanced laparoscopic proficiency among residents, clinical medical postgraduates, and OR nurses. The findings indicated that following laparoscopic simulation training, the fundamental laparoscopic skills of OR nurses were comparable to those of residents, demonstrating a similar level of competence. By improving virtual reality training efficiency in this field, our findings might transform surgical training across medical disciplines. Integrating these advanced methods not only aids new health care providers but also shows potential for enhancing patient outcomes and lowering the incidence of surgical complications. Moreover, our research underscores the need to integrate medical technology into training methods, thereby enhancing health care quality and overall safety. However, the following limitations still exist in this study: first, the sample size was relatively small; second, it was not validated in real patients; third, although this study showed that virtual simulation technology applied to clinical teaching can effectively improve trainees’ hands-on operation ability and comprehensive ability, the learning of theoretical knowledge needs to be further strengthened by multiperspective studies and reviews to continue to improve the comprehensive quality of trainees. In future teaching practice, the research team will further expand the scope of the study, increase the number of study participants, create other scientific parallel control groups, optimize more observation indices, further improve the teaching level and clinical surgical operation skills teaching ability of the clinical teachers through training, and continuously promote the teaching mode based on the virtual simulation laparoscopic system platform to facilitate the innovation of the standardized training mode of OR nurses.

### Supplementary Information

**Additional file 1: Table S1.** Camera navigation. **Table S2.** Peg transfer. **Table S3.** Coordination. **Table S4.** Fine dissection.

## Data Availability

All data generated or analyzed during this study are included in this published article.
