# Supplementary material for: Effect of virtual reality training to enhance laparoscopic assistance skills
Source: BMC Med Educ. 2024 Jan 4;24:29. doi: 10.1186/s12909-023-05014-5 (PMC10768454; doi:10.1186/s12909-023-05014-5)
Supplement: Supplementary file 1 — Additional file 1: Table S1. Camera navigation. Table S2. Peg transfer. Table S3. Coordination. Table S4. Fine dissection. [file 12909_2023_5014_MOESM1_ESM.docx]

**Table S1 Camera navigation**

| Result Parameter | Min | Opt.Min | Opt.Max | Max | weight |
| --- | --- | --- | --- | --- | --- |
| Total Time [s] | 0.00 | 0.00 | 30.00 | 60.00 | 15% |
| Misses [%] | 0.00 | 0.00 | 50.00 | 100.00 | 13% |
| Drift [mm] | 0.00 | 0.00 | 5.00 | 10.00 | 11% |
| Instrument Path Length [m] | 0.00 | 0.00 | 1.50 | 3.00 | 12% |
| Instrument Angular Path [degrees] | 0.00 | 0.00 | 360.00 | 720.00 | 12% |
| Tissue Damage [#] | 0.00 | 0.00 | 5.00 | 10.00 | 13% |
| Maximum Damage [mm] | 0.00 | 0.00 | 15.00 | 30.00 | 12% |
| Angled Horizon Error [degrees] | 0.00 | 0.00 | 0.00 | 90.00 | 12% |

**Table S2 Peg transfer**

| Result Parameter | Min | Opt.Min | Opt.Max | Max | weight |
| --- | --- | --- | --- | --- | --- |
| Total Time [s] | 0.00 | 0.00 | 200.00 | 300.00 | 8% |
| Left Instrument Path Length [m] | 0.00 | 0.00 | 2.00 | 4.00 | 4% |
| Left Instrument Angular Path [degrees] | 0.00 | 0.00 | 500.00 | 1000.00 | 5% |
| Left Grasps [#] | 0.00 | 5.00 | 7.00 | 16.00 | 5% |
| Right Instrument Path Length [m] | 0.00 | 0.00 | 2.00 | 4.00 | 4% |
| Right Instrument Angular Path [degrees] | 0.00 | 0.00 | 500.00 | 1000.00 | 5% |
| Right Grasps [#] | 0.00 | 5.00 | 7.00 | 16.00 | 5% |
| Minimum Switches Per Transfer [#] | 0.00 | 0.00 | 0.00 | 0.00 | 5% |
| Maximum Switches Per Transfer [#] | 0.00 | 1.00 | 2.00 | 10.00 | 5% |
| Mean Switches Per Transfer [#] | 0.00 | 1.00 | 1.50 | 5.00 | 5% |
| Standard Deviation of Switches Per Transfer [#] | 0.00 | 0.00 | 2.00 | 5.00 | 5% |
| Minimum Drops Per Transfer [#] | 0.00 | 0.00 | 0.00 | 0.00 | 5% |
| Maximum Drops Per Transfer [#] | 0.00 | 0.00 | 1.00 | 3.00 | 5% |
| Mean Drops Per Transfer [#] | 0.00 | 0.00 | 1.00 | 2.00 | 5% |
| Standard Deviation of Drops Per Transfer [#] | 0.00 | 0.00 | 2.00 | 3.00 | 5% |
| Peg Transfer Misses [%] | 0.00 | 0.00 | 25.00 | 50.00 | 5% |
| Grasp Misses [%] | 0.00 | 0.00 | 25.00 | 50.00 | 4% |
| Exceeded Grasp Transfer Misses [%] | 0.00 | 0.00 | 25.00 | 50.00 | 5% |
| Time Grasp with Left Instrument [s] | 0.00 | 0.00 | 100.00 | 150.00 | 5% |
| Time Grasp with Right Instrument [s] | 0.00 | 0.00 | 100.00 | 150.00 | 5% |

**Table S3 Coordination**

| Result Parameter | Min | Opt.Min | Opt.Max | Max | weight |
| --- | --- | --- | --- | --- | --- |
| Total Time [s] | 0.00 | 0.00 | 30.00 | 60.00 | 12% |
| Misses [%] | 0.00 | 0.00 | 50.00 | 100.00 | 10% |
| Left Instrument Path Length [m] | 0.00 | 0.00 | 1.50 | 3.00 | 10% |
| Left Instrument Angular Path [degrees] | 0.00 | 0.00 | 360.00 | 720.00 | 10% |
| Left Instrument Outside View [#] | 0.00 | 0.00 | 0.00 | 10.00 | 10% |
| Left Instrument Outside View [s] | 0.00 | 0.00 | 0.00 | 60.00 | 10% |
| Camera Path Length [m] | 0.00 | 0.00 | 1.50 | 3.00 | 9% |
| Camera Angular Path [degrees] | 0.00 | 0.00 | 360.00 | 720.00 | 9% |
| Tissue Damage [#] | 0.00 | 0.00 | 5.00 | 10.00 | 10% |
| Maximum Damage [mm] | 0.00 | 0.00 | 15.00 | 30.00 | 10% |

**Table S4 Fine dissection**

| Result Parameter | Min | Opt.Min | Opt.Max | Max | weight |
| --- | --- | --- | --- | --- | --- |
| Total Time [s] | 0.00 | 0.00 | 180.00 | 360.00 | 9% |
| Ripped or Burned Blood Vessels [%] | 0.00 | 0.00 | 25.00 | 50.00 | 7% |
| Energy Damage on Blood Vessels [%] | 0.00 | 0.00 | 50.00 | 100.00 | 7% |
| Ripped Small Vessels [%] | 0.00 | 0.00 | 50.00 | 100.00 | 7% |
| Burned Small Vessels [%] | 70.00 | 80.00 | 100.00 | 100.00 | 7% |
| Burned Small Vessels w/o Stretch [%] | 0.00 | 0.00 | 50.00 | 100.00 | 7% |
| Grasper Path Length [m] | 0.00 | 0.00 | 1.50 | 3.00 | 7% |
| Grasper Angular Path [degrees] | 0.00 | 0.00 | 360.00 | 720.00 | 7% |
| Grasper Outside View [#] | 0.00 | 0.00 | 0.00 | 10.00 | 7% |
| Grasper Outside View [s] | 0.00 | 0.00 | 0.00 | 60.00 | 7% |
| Cutter Path Length [m] | 0.00 | 0.00 | 1.50 | 3.00 | 7% |
| Cutter Angular Path [degrees] | 0.00 | 0.00 | 360.00 | 720.00 | 7% |
| Cutter Outside View [#] | 0.00 | 0.00 | 0.00 | 10.00 | 7% |
| Cutter Outside View [s] | 0.00 | 0.00 | 0.00 | 60.00 | 7% |
